# Supplementary figures and images for: Comprehensive characterization of mitochondrial bioenergetics at different larval stages reveals novel insights about the developmental metabolism of Caenorhabditis elegans
Source: bioRxiv. 2024 Jun 30:2024.06.26.600841. Preprint. [Version 1] doi: 10.1101/2024.06.26.600841 (PMC11230424; doi:10.1101/2024.06.26.600841)

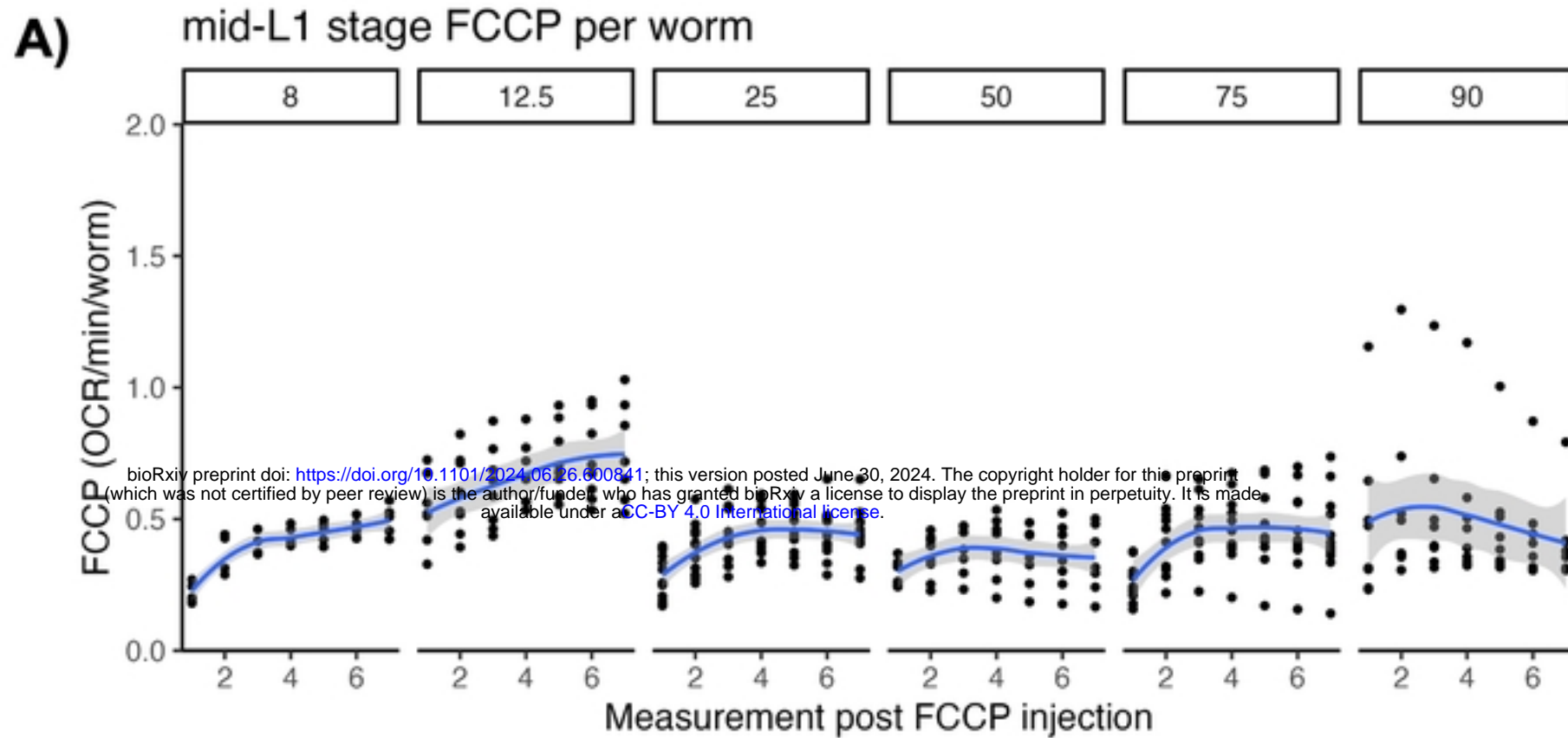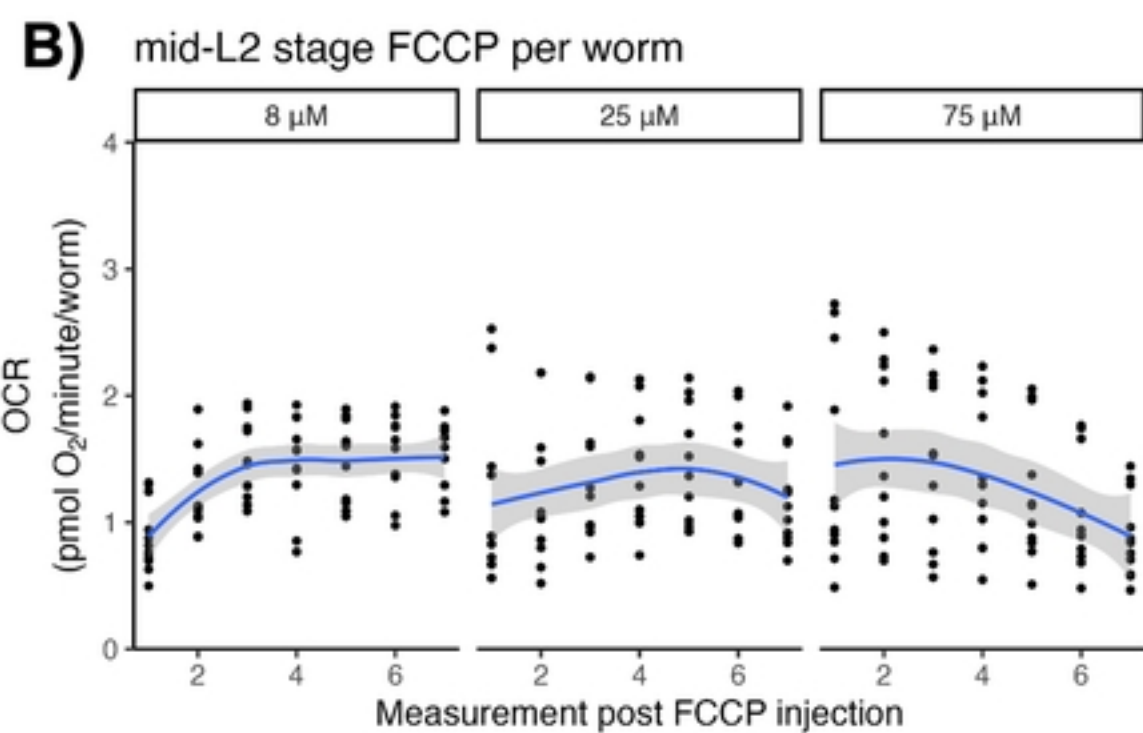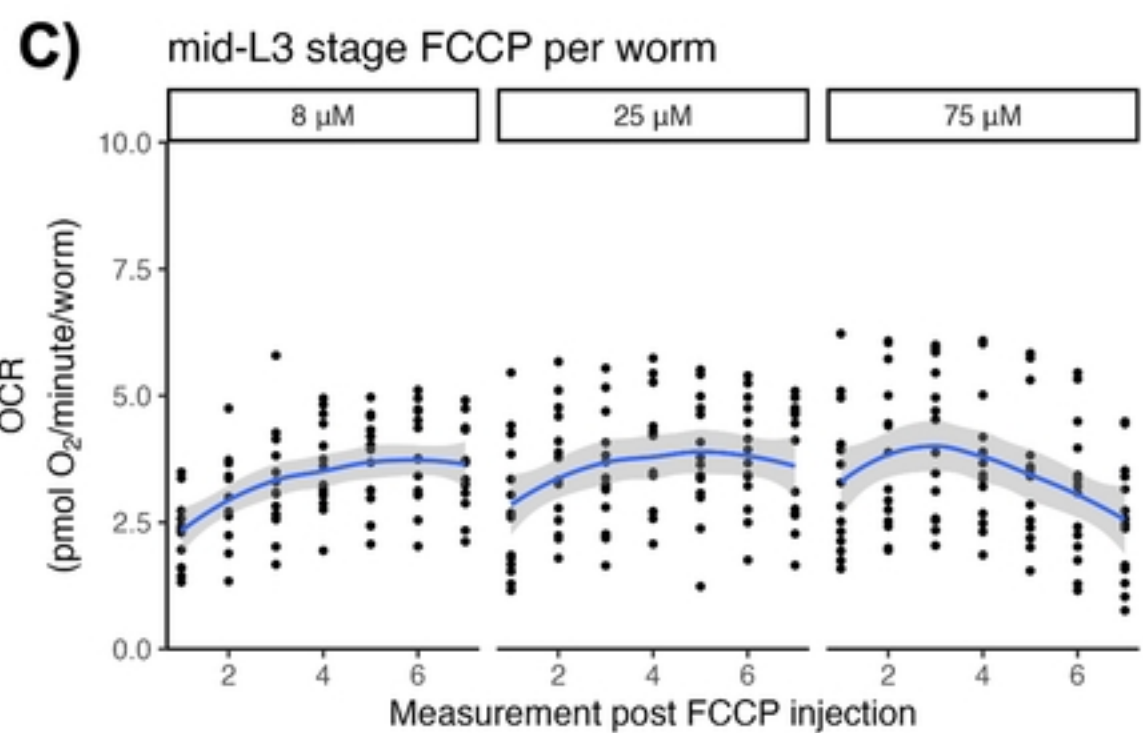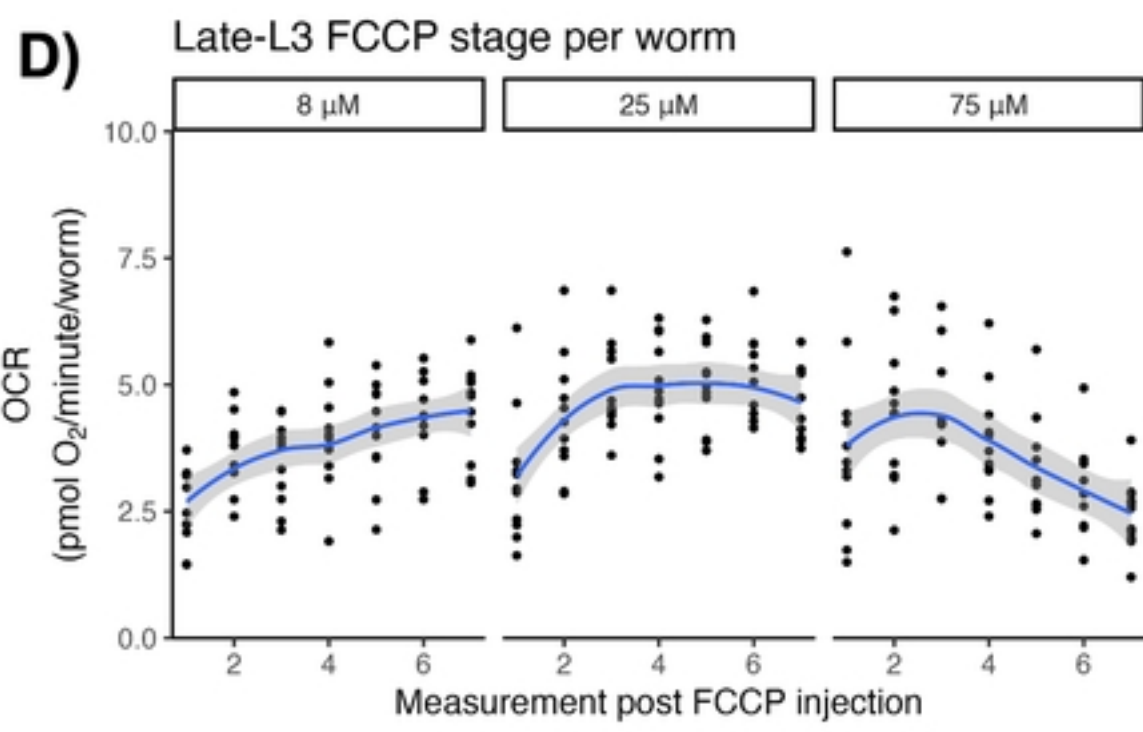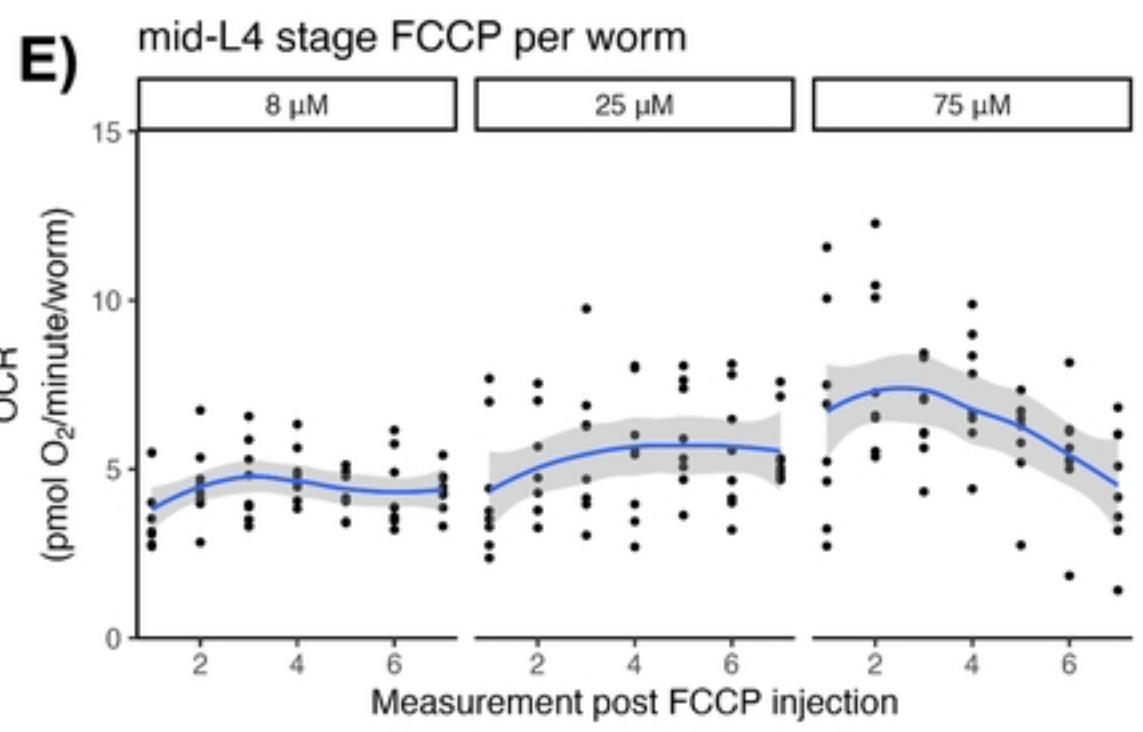

Figure S2

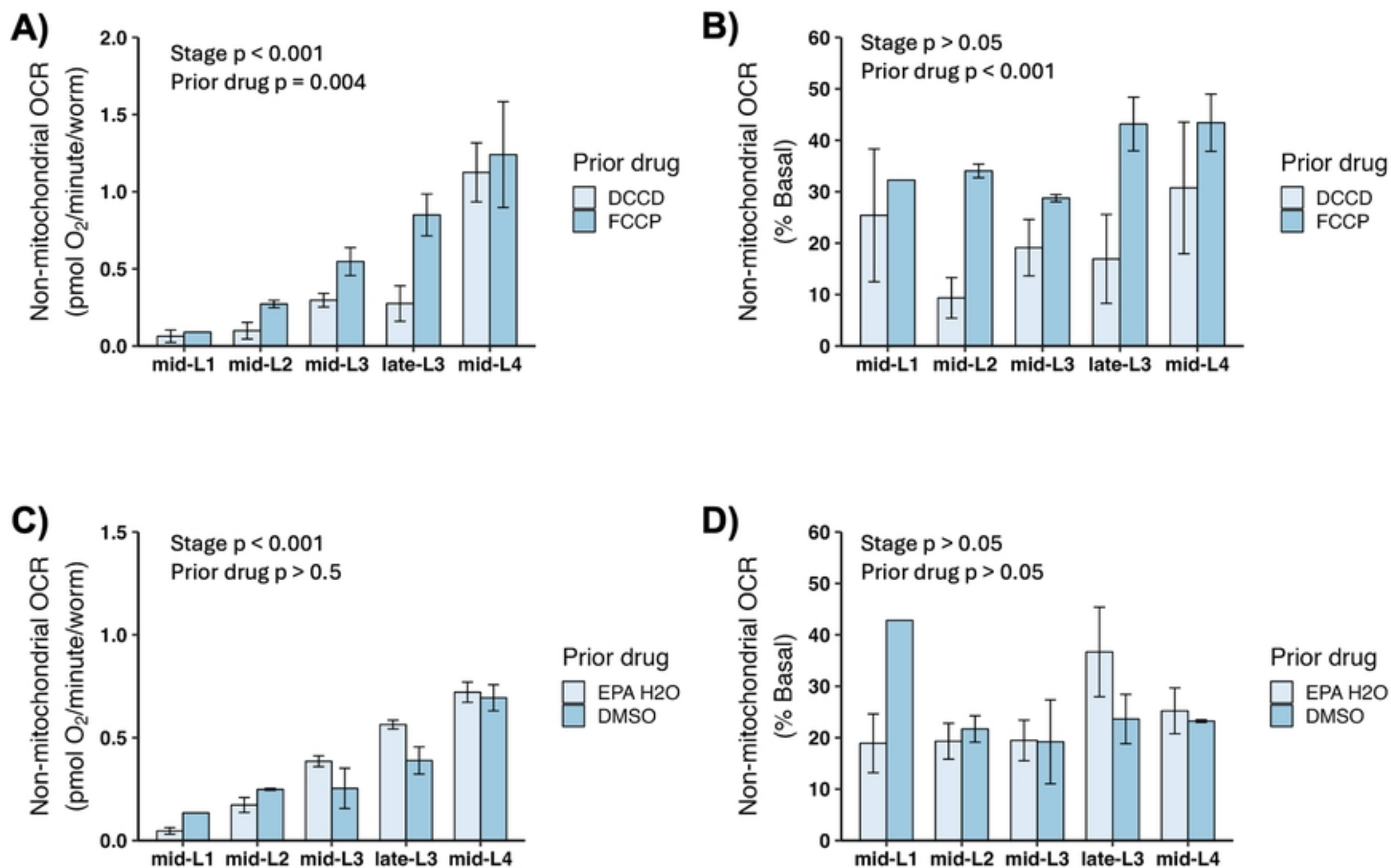

Figure S5

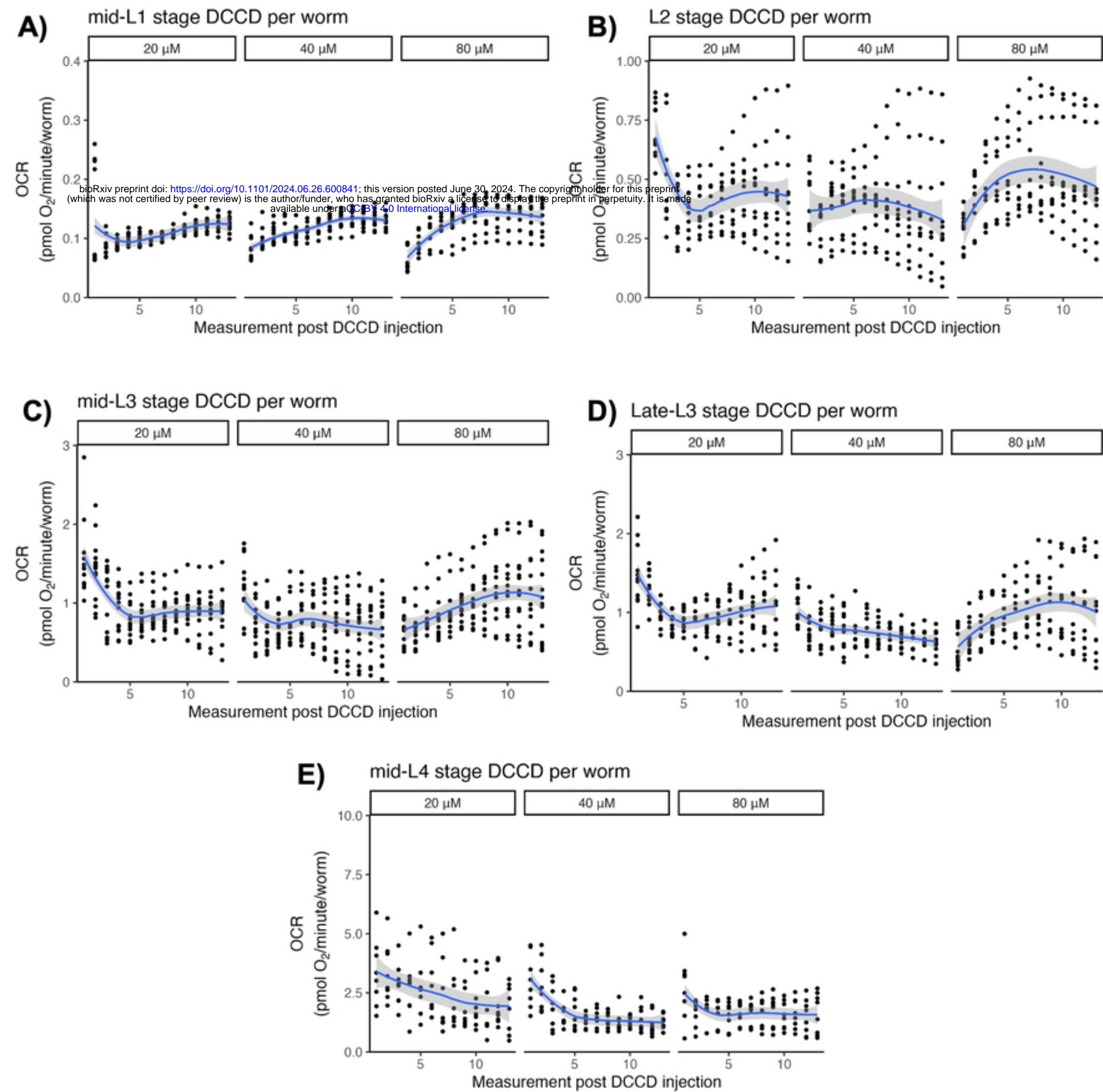

Figure S3

Supplement: Supplement 1 — S2. Figure S2 shows oxygen consumption rates at the L1 (panel A), L2 (panel B), mid-L3 (panel C), late L3 (panel D), and L4 (panel E) stages at different timepoints after the injection of different concentrations of FCCP, normalized per worm. Blue line represents local polynomial regression fitting of the data for visualization with 95% confidence interval using the geom_smooth function in ggplot2 package in R version 4.2.1. Figures A-E represent data across 1–4 biological replicates with 3–6 technical replicates (L1 – 1–3 biological reps with 4–5 technical replicates; L2 – three biological reps with 3–5 technical replicates; L3 – four biological reps with 3–5 technical replicates; Late-L3-three biological reps 3–5 technical replicates; L4 – two biological reps with 3–5 technical replicates). S3. Figure S3 shows oxygen consumption rates at the L1 (panel A), L2 (panel B), mid-L3 (panel C), late L3 (panel D), and L4 (panel E) stages at different timepoints after the injection of different concentrations of DCCD, normalized per worm. Blue line represents local polynomial regression fitting of the data for visualization with 95% confidence interval using the geom_smooth function in ggplot2 package in R version 4.2.1. Figures A-E represent data across 2–4 biological replicates with 3–6 technical replicates (L1 – two biological reps with 4–6 technical reps; L2 – three biological reps with 3–5 technical reps; L3 – four biological reps with 3–5 technical reps; Late-L3-three biological reps with 3–5 technical reps; L4 – two biological reps with 3–5 technical reps). S5. Figure S5 shows the effects of including DMSO solvent vs EPA water, prior FCCP injections, and prior DCCD injections on sodium azide-mediated inhibition of oxygen consumption rates at different larval stages. Non-mitochondrial OCR normalized per worm and percent basal after injection with FCCP and DCCD. n = 1–4 biological replicates, p-values from two-way ANOVA. [file NIHPP2024.06.26.600841v1-supplement-1.pdf]
